# Controlling sickle cell disease in Ghana - ethics and options

**DOI:** 10.4314/pamj.v10i0.72223

**Published:** 2011-10-03

**Authors:** Ama Kyerewaa Edwin, Frank Edwin, Victor Etwire

**Affiliations:** 1 Ethics Unit, Medical Directorate, Korle Bu Teaching Hospital, Accra, Ghana; 2 National Cardiothoracic Center, Korle Bu Teaching Hospital, Accra, Ghana; 3 Pediatric Surgery Unit, Korle Bu Teaching Hospital, Accra, Ghana

**Keywords:** Ethics, sickle cell disease, prenatal diagnosis, selective abortion, options

## Abstract

Sickle Cell Disease (SCD) is a significant public health burden in Ghana. Recent studies indicate that 2% of Ghanaian newborns are affected by SCD; one in three Ghanaians has the hemoglobin S and/or C gene. As a means of controlling the disease, some authorities have recommended prenatal diagnosis (PND) and selective abortion. In the current era, SCD has a good prognosis and fairly reasonable quality of life. Advances in bone marrow transplantation have shown the disease is curable in selected patients. PND and selective abortion therefore raises a myriad of ethical dilemmas which are considered in this review. In the light of the demonstration of improved prognosis in recent times, PND and selective abortion appears to be applying capital punishment to the unborn child for “crimes” only the parents can be responsible for. In this review, we recommend control of SCD on three levels – preconception genetic testing and strategic reproductive choices, PND and education for carrier parents, and holistic management of persons with SCD. We emphasize the critical importance of self-management, especially self-awareness, in assuring a good quality of life for persons with SCD. We believe such an approach is cost-effective, and consistent with sound ethical principles and good conscience.

## Background

Sickle Cell Disease (SCD) refers to a group of conditions characterized by the presence of hemoglobin S (HbS) and one other abnormal hemoglobin. According to the World Health Organization (WHO), 5.2% of the world's population and over 7% of pregnant women carry an abnormal haemoglobin gene [1]. SCD is most prevalent in Sub-Saharan Africa where malaria is endemic. The genotypes characterized by Hb SS and Hb SC are dominant in the sickle cell disease population of Ghana and form the basis of this communication. Reports from Ghanaian studies indicate a carrier rate in the population of 30% whereas 2% of Ghanaian newborns have sickle cell disease [2].

### Clinical and social relevance

Like many other chronic diseases, SCD has a huge psychosocial burden. Sickle cell anemia is a severe recessive genetic disorder; the homozygous state results in an abnormal hemoglobin that is prone to polymer formation under cellular deoxygenation. The polymerized hemoglobin reduces red blood cell deformability and causes “sickling” of erythrocytes within capillaries and end-arterioles. The resulting microcirculatory obstruction gives rise to vaso-occlusive crises which may occur repeatedly in the presence of certain triggers. Clinically, the disease is characterized by chronic hemolytic anemia interspersed with episodes of acute vaso-occlusive sickling crises. In the “stable state”, most patients with Hb SS have a steady state hemoglobin level of 8g/dl. Hb SC patients tend to have a higher steady state hemoglobin level of about 11g/dl but more frequent tissue infarctions (e.g. avascular necrosis of the femoral head). Sickling crisis results from episodes of increased red blood cell sickling and capillary sludging causing poor organ perfusion. Severe pain is the most common manifestation of sickling crisis. Crises are often precipitated by dehydration, infection, exposure to extremes of temperature (fever or cold exposure), and hypoxia. Periodic crises, the direct and indirect costs of frequent hospitalizations, the economic burden of SCD, and the early deaths that sometimes occur can lead to poor family relationships. This is made worse by the limited and often inaccessible formal social support structures that can help patients and families cope better with the psychosocial burden of SCD. The recurrent crises frequently cause absenteeism from school and work, eventually leading to school dropouts and job losses. This in combination with the frequent stigmatization and discrimination people with SCD face in Ghana can lead to a further sense of isolation from family and society [3]. The stigmatization and discrimination associated with SCD is such that patients sometimes lose their accommodation. One young man and his family were ejected from their accommodation when his landlord got to know that he has Sickle Cell Anaemia [4]. Thus the psychosocial burden of SCD is not insignificant.

### Prognosis of SCD

The prognosis for SCD is variable and reflects the interaction between individual biology and environmental factors. In a prospective study of the natural history of sickle cell disease, 3,578 patients ranging from newborns to those 66 years old were followed up for 18,356 patient-years [5]. The investigators found the average pain rate to be 0.8 episodes per patient-year in hemoglobin SS disease, and 0.4 episodes per patient-year in hemoglobin SC disease. In the study period, 39% of patients with hemoglobin SS disease had no episodes of pain, whereas 1% had more than six episodes per year. The 5.2 % of patients with 3 to 10 episodes per year had 32.9 % of all episodes. Furthermore, 80 % of hospital admissions for painful crisis occurred among 20% of patients. Among patients with sickle cell anemia who were more than 20 years old, those with high rates of pain episodes tended to die earlier than those with low rates [5,6]. The findings suggest that the medical profession's perspective of the clinical impact of SCD (derived mostly from hospital admissions) is likely to have been biased by the relatively small group of patients with frequent painful episodes. Increasingly, most people with SCD are reaching adulthood due to greater medical understanding and more effective intervention. Quinn and coworkers [7] studied the Dallas Newborn Cohort to estimate contemporary 18-year survival for newborns with SCD. These authors documented that most children with hemoglobin SS disease (93.9%) and nearly all children with milder forms of SCD (98.4%) survived to become adults [7]. Additionally, researchers are showing that bone marrow transplantation cures the disease in selected patients [8]. Multicenter trials indicate that hematopoietic stem cell transplantation in SCD is associated with an excellent outcome with overall and event-free survivals of approximately 93% and 86%, respectively [9]. Although these investigators limited their work to selected patients, it is envisaged that a cure shall be made widely available to all patients if the current momentum of research is maintained.

By all indications, the prognosis for SCD has improved markedly over the past two to three decades. How much of this information is made available to mothers with “at risk” fetuses after PND is debatable. Recent evidence [5,7–9] shows that in the current era SCD has a good prognosis in terms of survival and quality of life and a cure for the disease may soon be widely available. Although such high technological and expensive care may not be accessible to patients with SCD in a limited resource country like Ghana, patients can still have a good prognosis with the appropriate medical and psychosocial support.

### Prenatal diagnosis and selective abortion

Because it is a significant public health burden in Ghana, it is desirable to control and prevent SCD. As a means of controlling the disease, some workers have recommended prenatal diagnosis (PND) and selective abortion of affected fetuses in addition to preconception/premarital genetic testing and strategic reproductive choices, PND and education for carrier parents, and holistic management of persons with SCDIn particular, “some proponents of the Human Genome Project from the fields of science and bioethics argue that in a world of limited resources, we can reduce disability-related expenditures if all diagnoses of fetal impairment are followed by abortion” [10]. Selective abortion refers to an abortion where the parent(s) choose(s) to terminate a desired pregnancy because of a genetic disorder (or some other undesirable fetal characteristic). This is distinguished from elective abortion which is the planned termination of an unwanted pregnancy. This report reviews the ethical dilemmas in PND and selective abortion as a policy in Ghana and suggests options for controlling SCD on three levels. This is particularly important in Ghana, which, until the development of the Draft Strategic Framework for the Management, Prevention and Control of Sickle Cell Disease in Ghana in March 2010 had no clear strategic framework for managing and preventing SCD [3].

## Ethical issues in prenatal diagnosis and selective abortion

Prenatal diagnosis was introduced in the 1970s on the grounds that genetic counseling removes anxiety of mothers, reduces the number of children born with certain diseases, and increases the economic advantages to society [11]. Although PND and selective abortion have some benefits such as the availability of information about the unborn child to expectant parents and doctors, alleviation of anxiety in “at risk” pregnancies and the avoidance of unnecessary terminations [11], a number of ethical dilemmas arise. Some of these ethical issues such as the technique of PND and the time of gestation it is offered, the moral justification of PND and selective abortion, the rights of parents against the rights of society regarding decisions about pregnancy, the risks and benefits of PND and selective abortion, and the ethics of aborting a genetically-disabled fetus shall be considered.

### Issues relating to the technique of PND

Commonly, PND relies on amniocentesis or chorionic villus sampling (CVS), both of which are invasive, to obtain fetal material for testing. Both are associated with a 1-3% risk of miscarriage relating to the procedure. CVS can be performed earlier in pregnancy than amniocentesis, however it carries a higher risk of fetal harm. Potential mothers would prefer an earlier diagnosis to minimize the psychological impact of abortion. This however may come at the increased risk of inducing abortion of a genetically normal child which creates an ethical conundrum.

The psychological burden of selective abortion is less the earlier in gestation the termination is performed; but at what stage in the life of a fertilized ovum is it considered a human being? Is the embryo a human being or not? We agree with Tangwa that “embryos do have human status and a morally significant line cannot be drawn between human embryos and other human beings” [12]. Does the unborn child have rights to protection, nutrition, and life? Yes, as a human being, it does have rights since the fetus has the same moral status as all existing humans [12].

Some geneticists are of the opinion that patients should agree to an abortion, if the test result turnout positive, before being offered PND. What if the mother undergoes PND but chooses not to have an abortion? Should such mothers be “allowed” to have PND? Does the mother always understand the consequences of the information given after the testing? It is generally agreed that in order not to compromise the patient's autonomy, genetic counseling should be non-directive; but is this realistic? Genetic counselors and others have expressed doubt about whether it is theoretically or practically possible to have non-directive counseling. The very nature or process of prenatal counseling, some argue, presupposes an implicit bias to abort selectively any fetus deemed “defective” [13].

Program directors implicitly tend to assess the success of PND schemes by an undeclared preventive index: the number of “abnormal babies” prevented from coming to birth as a fraction of genetically abnormal babies detected by PND. How then can genetic counseling be non-directive and offer mothers’ free choice? The non-directiveness of genetic counseling is underlined by the fact that the very act of giving information on fetal defects and disabilities within a medical setting with abortion being offered as an alternative makes abortion itself a “live option” [13]. More often than not, pregnant women undergo these tests without being aware that they may opt out of it especially when obstetricians suggest prenatal testing as part of routine anternatal care. For those providing genetic counseling to pregnant women and or expectant parents, how they communicate the importance of prenatal testing and the implications of its outcome for the parents to be, the family and the larger society plays a major role in what parents do after prenatal testing. Despite the fact that genetic counseling ought to be non-directive, the practice of many professionals is such that it does not legitimize the choices of those who choose to continue with the pregnancy of an affected child. Even when pregnant women have made a choice, they still have their judgments questioned by clinicians. As one woman puts it: “As I was examined and interviewed by several different disciplines, I was left with the impression that continuing a pregnancy (of a fetus with spina bifida) such as mine was an unusual thing to do. It seemed as though every time I turned around another physician was asking me whether or not anyone had discussed my options with me”. “Options” has clearly become a euphemism for “abortion” [13]. It seems therefore that for many people, counseling itself offers a message.

As several scholars have indicated [14], the resources allocated to genetic counseling and testing is largely premised on the notion that most people will terminate the pregnancy of an affected fetus. Hopefully, practical measures put in place to ensure that genetic counseling is done in a non-directive manner can go a long way.

### Prenatal diagnosis and selective abortion – Capital punishment for parental “crimes”?

The current law on abortion in Ghana legalized termination of pregnancy on the basis of a serious physical abnormality or disease [15]. Although the law does not define “serious”, it allows termination on the basis of risk to the mental health of the pregnant woman. The law therefore seems to support selective abortion on the basis of maternal psychological distress. Whereas some see this kind of liberal policy on selective abortion as a moral gain that avoids suffering by getting rid of abnormal fetuses, others see in this a resurgence of eugenic philosophy that promotes “ante-natal euthanasia” [16]. If the sole aim of PND is to identify and treat abnormalities prenatally or postnatally, there would be very little objection, if any at all. Unfortunately, most PND is done to identify and selectively abort “abnormal” babies, creating ethical dilemmas. It would appear as though the unborn child with a genetic “abnormality” has been sentenced to capital punishment for a “crimes” he/she did not commit. The question then is: whose interest does PND serve, is it the unborn child, the parents, the siblings or society? If PND leads to prenatal or postnatal treatment, then obviously, the interests of the child would have been served since the aim will be to give that child a better life. In as much as most people will prefer to have a healthy child, the interests of this unborn child vis-à-vis having a life at all should outweigh what Aksoy refers to as “comparatively trivial interests, for almost any interest is trivial compared to life” [17]. The use of the word “trivial” is relative here and does not mean that those who opt for PND and selective abortion do it in a cavalier manner. Despite the fact that a pregnant woman's autonomy would be more infringed by having no option at all, the complex and difficult challenges that mothers face as they make decisions regarding the termination of a genetically defective child is anything but trivial.

The psychological impact of abortion following prenatal diagnosis is well documented [18]. These include emotionally devastating consequences for parents as well as living children, psychosomatic disorders, guilt, anger, depression and other emotional painful conditions and prolonged periods of adjustment. As one author puts it; “For many women and their partners, the decision to terminate a pregnancy after a prenatal diagnosis of a serious genetic defect can be harrowing, often coming after a painful assessment of their own emotional and financial resources” [19].

If SCD was not compatible with life, or no satisfactory treatment existed, the ethical dilemma would be less. However, not only is SCD compatible with life but there are numerous accounts of sufferers surviving with a good quality of life and making very significant contributions to society. One young Ghananian professional career woman with sickle cell anemia put it this way:

“*Everybody should be given a chance at life because you never know. I don't believe in aborting your baby if you think that child has SCD. I know of other people who have survived and are doing well and contributing to society. So give them a chance. When you abort the child, you have not even given him/her the chance to live*” [20].

Some workers have noted that although prenatal screening is purportedly about offering an informed choice, nonetheless it is impossible to avoid the implicit assumption about the undesirability of the existence of people with such a condition and of giving birth to and bringing up such people. The promotion of prenatal screening programmes and the subsequent abortion of affected fetuses cannot help but carry a moral message about the worth of the lives of those individuals who have the disorders [21].

### Pregnancy Decisions – The rights of parents versus the rights of society

For women or couples whose PND tests show abnormal results, a number of moral, religious and social factors and pressures determine the choices made. In addition, the emotional consequence of deciding to abort a child with SCD, a condition which is compatible with life at reasonable quality, especially when that child was wanted can have a heavy toll of guilt on pregnant women and couples.

In balancing the rights of parents against the rights of society in regard to decisions about the continuation or termination of a pregnancy, can a mother who is unwilling to abort an affected child be “forced” by society or required by law to terminate that pregnancy in order to spare society the economic and social burden of an affected child? Are we so economically challenged that we must base the decision of life or no life on economic savings to society? By suggesting abortion of SCD fetuses, are we then basing the choice predominantly on economics? Although there may be some economic advantages to society, surely, abortion is no remedy for economic trouble.

Certainly, such a move by society can be seen as marking the rebirth of a eugenic movement. The long arm of the law may not reach this far but the social pressures and disdain that may be heaped on the mother or couple for knowingly giving birth to an affected child may be strong enough to let some terminate a pregnancy they would otherwise have kept. Consequently, in balancing parental and societal interests, the interests of the unborn child must also be factored in. Is it in this child's best interest to be denied a life because of a diagnosis of SCD? Are the disabilities associated with SCD so terrible that a prenatal diagnosis and pregnancy termination would be a preferred alternative for the child who has been diagnosed prenatally with this condition? In our view, the argument that prenatal diagnosis and pregnancy are justified on the basis of the best interest of the child is not tenable [22].

Doubtless, SCD imposes significant psycho-social and economic burdens on affected families. Though the condition can be satisfactorily managed, families often face enormous challenges characterized by repeated disruptions in family routines and competition with income-generating family endeavors. The work of Famuyiwa and Akinyanju [23] showed a significant correlation between total burden scores on families with SCD and frequency of crisis. Ohaeri and Shokunbi [24] reported that objective burden indices were significantly higher for SCD in crisis compared to the steady state. Furthermore, the financial burden of SCD in crisis was significantly higher than the burden of disruption of family routines and that nearly 58% of all caregivers experienced little or no difficulty coping with SCD. They suggested that the psychosocial burden of SCD can be significantly reduced by controlling the frequency and duration of crisis, as well as providing adequate information and socioeconomic support to families. The primary determinant of family burden in sickle cell disease (which appears to be primarily economic) is therefore the frequency of sickling crisis. By instituting the appropriate measures discussed under holistic management, the financial burden can be significantly reduced.

## The risks versus benefits of prenatal diagnosis and selective abortion

There is no doubt that a technology that is relatively non-invasive with a primary purpose of diagnosing and treating conditions prenatally or postnatally will be welcomed by all. Unfortunately, PND rarely leads to fetal treatment. On the contrary, many normal fetuses are affected negatively by chorionic villus sampling used for PND. Although CVS appears to pose little risk to the woman, there is a 3.2% procedure-related fetal loss; with amniocentesis this rate is less than 1%. By undergoing CVS for PND, a woman accepts a one in thirty chance of miscarrying what is probably a normal fetus. This aside, the occurrence of limb reductions in genetically normal babies is attributed to CVS [25].

Although part of the goal of the Human Genome Project was said to be the cure or treatment of genetic diseases [14], to date very few therapies that will help those with genetic conditions have been developed. Rather than developing treatments for most of the conditions in which a specific gene has been found, research has focused on the development of prenatal tests that gives information about the future child with little if any being done to find treatment for those living with the conditions now.

Furthermore, the widespread acceptance of selective abortion has decreased the motivation for research on cures for genetic disorders with scientists abandoning research on cures for genetic disorders in favor of selective abortion. For example, in the 1960s, up to three times as many people were working on a cure for Tay-Sachs disease than currently. The emphasis has shifted to PND with selective abortion in case of a positive test [26]. Similarly, with the availability of PND for Huntington's chorea in the early 1980s, “funds began to disappear for research to find a cure” [27]. It is likely that with widespread availability of PND and selective abortion for SCD, a similar fate will befall SCD. This will be tragic indeed considering the fact that a cure has been demonstrated for selected patients with SCD in centers in the developed world. What remains for researchers to accomplish is to make the treatment widely available. If the current momentum of PND and selective abortion overturns the impressive gains already made in finding a cure for SCD, we would have written another regrettable chapter in the history of mankind.

## Society's ethics regarding selective abortion

One of the justifications for access to abortion when PND first became available in some jurisdictions was to avoid the birth of a child with serious disability. According to some disability experts, using PND in this manner is discriminatory against people with disability and implies that their lives are considered less valuable than those without disability [28]. In any case, there are several people living with disabilities (some very debilitating) who have contributed more to society than many with no such disabilities. What if a genetic test had indicated that Stephen Hawking's parents would have a child who will later develop amyotrophic lateral sclerosis? Put another way, would a mother abort her fetus if she knew the unborn child would become the modern-day Albert Einstein despite a serious disability?

As Asch eloquently put it:

“*People with disabilities do not merely take from others, they contribute as well – to families, to friends, to the economy. They contribute neither in spite of nor because of their disabilities, but because along with their disabilities come other characteristics of personality, talent, and humanity that render people with disabilities full members of the human and moral community*” [10].

In the current era, SCD has a reasonable life expectancy and acceptable quality of life in spite of the challenges in caring for persons with the disease. When society chooses selective abortion of fetuses with SCD, it has chosen the convenient option, not necessarily the best one. Unfortunately, that choice raises a myriad of ethical concerns. What kind of society upholds animal rights and animal conservation but aborts its babies on the grounds of SCD. The use of animals in laboratory research has received heated criticism in spite of the fact that the British Royal Society argues that virtually every medical achievement in the 20th century relied on the use of animals in some way [29]. A number of scientists and animal rights organizations have questioned the legitimacy of animal testing, arguing that it is cruel, and constitutes poor scientific practice. Some insist that animals have intrinsic rights not to be used for experimentation (and euthanized afterward) [30]. Organizations such as the Royal Society for the Prevention of Cruelty to Animals (RSPCA), have even gone further to argue that the lives of laboratory animals have intrinsic value and so even a painless death can constitute a cost. The RSPCA makes the strong point that “death should be considered a harm and should be given serious weighting since presumably an individual animal that is not suffering substantially would not actually choose to give up his or her own life” [31]. The RSPCA's position is a reminder of a fundamental ethical principle: “first do no harm”, which proponents of selective abortion of SCD fetuses have conveniently forgotten. We must ask if the fetus with SCD or indeed the person with SCD suffers substantial physical and emotional pain so incapacitating for them to consider giving up their own lives. The scientific community generally upholds the idea that animal life should not be taken wantonly. In the light of these arguments, when society condones selective abortion of its fetuses with SCD, the implicit message is that somehow the lives of laboratory animals have more intrinsic value than the lives of persons with SCD. Surely, it is inconceivable to compare human life with that of laboratory animals, but from the perspective of scientists who live with SCD and still manage to contribute their quota to the human family, even to its scientific knowledge base, society's ethics can be described as confusing at best.

## Controlling sickle cell disease-The options

From the foregoing, PND and selective abortion is a questionable option in controlling SCD in Ghana. As one prominent Ghanaian hematologist puts it:

*“I would not advocate for PND and selective abortion. You would not know what or who you are aborting”* [32]. We believe SCD can be controlled on three levels that are more consistent with ethical principles and good conscience. These are preconception genetic testing and strategic reproductive choices, PND and education for carrier parents, and holistic management of persons with SCD:

### Preconception genetic testing and strategic reproductive choices

Pre-marital genetic testing for the hemoglobin S or C gene equips individuals with knowledge of their carrier status before entering a relationship with the view to having children. For this to be effective, it should be done before emotional bonding is advanced between two people in a relationship. It is important for communities to educate their members on SCD and its psychosocial issues so that carriers especially are given adequate information to make strategic reproductive choices. Two carriers may decide to go ahead into a relationship but their choice should be based on the full knowledge of the issues at stake. We do not prohibit marriage between carriers of the sickling gene. We believe that the ethical dilemmas and accompanying psychosocial burdens are less when two carriers put off a marriage than when they have to make a choice between abortion of a SCD fetus and having to face the psychosocial issues of caring for a SCD affected child. Preconception genetic screening and counseling should be the main focus of efforts at controlling SCD in developing countries because screening is relatively cheap and far less invasive that PND. Besides, the psychological and socioeconomic issues at stake are far easier to manage than when a couple must decide on PND and selective abortion. Though this aspect of control of the disease has not been given enough emphasis in Ghana, prevention of the disease through public education, awareness of one's carrier status and genetic counseling regarding reproductive choices is certainly a better ethical and economic option than prevention through PND and selective abortion of affected fetuses.

Where prevention of SCD is concerned in Ghana, our best approach should be genetic counseling and screening at all levels and age groups. Primary prevention can be achieved through population screening with Hb electrophoresis because of the high carrier rate of 30% of HbS or C in Ghana. Although it is costly, such a strategy will prove cost effective in the long term and should form part of the basic health services in Ghana [32]. This is in line with the principles of genetic screening for public health purposes which “tend to be most effective when it serves a clear population goal, has a healthy ratio of overall economic and social benefits to costs, entails a just use of resources, and is acceptable to the population it targets” [33]. It is difficult to justify the expense some African governments have made in providing facilities for PND [34] and selective abortions when the far simpler means of preconception genetic testing and counseling has been overlooked.

One may wonder whether a person with SCD would want the right to screen a future partner to know whether his/her own children were likely to suffer the same difficulties that they themselves have suffered? One patient indicated that before she got into any serious relationship, she found out whether the potential partner was a carrier of HbS gene or not. This is because she did not want any of her children to go through what she goes through [20]. The fact that patients would rather not have children with SCD in no way undermines the value of the lives of people with SCD. In as much as it is better to prevent certain common conditions like hypertension than to manage its sequelae, no one, to the authors’ knowledge, has proposed that all those diagnosed with hypertension should be killed so that families and societies can avoid the psychosocial and economic burdens associated with managing hypertension and its sequelae.

### Education for carrier parents

The greatest ethical challenge and dilemma occurs at this level. The options are however clear. When potential parents are ignorant of their carrier status, there are no issues until birth. The pregnancy proceeds naturally to birth or to fetal loss as the case may be. The real problem occurs when the potential parents know they are both carriers. Should they undergo PND? We have already mentioned the benefits of PND in these instances – the relief of parental anxiety and preparation for caring for an affected child are ostensibly the most important. It should be kept in mind though that PND is not without risk to the fetus, who may be genetically “normal”. But what if the fetus tests positive for SCD after PND? We do not favor selective abortion of SCD fetuses because we are convinced of a far better prognosis for most affected children in the current era. We believe that the genetic makeup of a fetus should not have the final say of its destiny. The test results should equip parents with information to prepare to care for their child. They need relevant and current information on how to care for their child through the various stages of its life. Especially relevant is information on how to avoid the triggers of sickling crises since outcomes are heavily dependent on the severity and frequency of crises [5]. In this regard, neonatal screening must also become part of the basic health service available in Ghana. The purpose of neonatal screening for SCD is to be able to offer optimal management from early infancy. It is noteworthy that in-line with this, the Ghana Health service (GHS) has taken the step towards a nationwide neonatal screening program [3]. However, the GHS needs to go beyond adopting this as a strategy to actually implementing it nationwide. Currently, only a few tertiary centres provide neonatal screening consistently.

Association with families and support groups who have successfully cared for children with SCD is helpful and we recommend it. Families need to re-organize their environments to prevent extremes of temperature, malaria and bacterial infections. Families need to determine the availability of appropriate healthcare facilities in the locality where they can obtain adequate care for their child. Vaccinations for common bacterial infections like the pneumococcus are recommended. It is our conviction that the good results obtained by others [7] caring for children with SCD is reproducible in Ghana with meticulous attention to the care of children with SCD.

### Holistic management of persons with SCD

Persons born with SCD have special needs and require a comprehensive care profile organized into medical and non-medical services, and self-management strategies. In order to optimize health care for people with SCD, a holistic approach that involves a multi-disciplinary team of well-trained professionals, and a well-defined social support system that meets the physical, emotional, psychological as well as the financial needs of patients with SCD is needed [35].

A comprehensive medical care program is best coordinated by a hematology team. It focuses on providing general pediatric and adult medical care and specialist care when the need arises.

Pain management is a vital part of medical care. Pain management is based on avoiding triggers of vaso-occlusive crisis, the common antecedent of painful episodes. Common analgesics used include acetaminophen and non-steroidal anti-inflammatory drugs. Ensuring adequate hydration is vital to successful and early alleviation of pain. The use of opiates is kept to a minimum and must be avoided as much as possible [36]. Preventive care involves immunizations against common pathogens and prophylactic penicillin. Blood-bank support is crucial as SCD patients tend to have a higher requirement for blood and blood products due to chronic hemolysis with superimposed hyper-hemolytic crises. Nutrition is important and supports immune function and proper development. Folic acid and vitamin supplements are useful adjuncts.

Non-medical services include psychotherapy (for psychological problems relating to school, work, and family), physiotherapy for joint problems, patient-parent information and peer-parent support groups, job training and vocational services.

The most important factor influencing the quality of life in SCD patients is self-management. Self-management interventions encourage acquisition or improvement of effective coping strategies to alleviate symptoms and achieve a better quality of life. Many patients with SCD successfully manage their disease in the outpatient setting without the need for frequent health care utilization and do not require frequent hospitalizations and/or emergency department visits. Potentially, much could be learned from this group in terms of living productively with SCD. A recent workshop involving such a group identified key strategies for effective self-management [37]. The most important strategies identified were self-awareness, emotional/spiritual support, career selection and success factors, nutrition, advocacy, knowledge, appropriate physical exercise, and complementary/alternative medicine. Self-awareness, the most vital aspect of self-management, is a sense of being aware of certain behaviors that promote or diminish health. It includes the practice of journaling and being in tune with the body to recognize overexertion, necessity for rest, and onset of illness. Journaling is a term coined for the practice of keeping a diary or journal that explores thoughts and feelings surrounding the events of one's life [38]. Journaling helps SCD patients to understand what type of activities trigger episodes of illness or pain [37]. With these strategies, persons with SCD are capable of achieving a near-normal life span with a reasonable quality of life.

The options offered for controlling SCD on these three levels provide avenues that we believe are ethical, cost-effective, and fair to all parties.

## Conclusion

Scientists have a moral and ethical responsibility to guide society with the insights that scientific knowledge affords. The prominence science has gained in modern society is summed up by Felix Konotey-Ahulu, world-renowned authority on SCD:

“*Scientists now determine the quality of life that society should allow, and they programme society carefully and insidiously to accept their guidance on these matters. History shows that often a few pressure groups, or even a single person, can decide what is good for the whole society. Nazi tribal society decided to get rid of Hebrew genes and programmed people to accept the plan as good for society. Now we must be careful not to allow scientific pressure groups whose only qualification is that they can see a particular gene to make us think that they are also qualified to tell us who should be allowed to survive and who should be aborted*” [39].

We wish to emphasize that biology is not synonymous with destiny. Human history shows that destiny is shaped not only by our genetic make-up but probably more importantly by our environment and training. The genetic makeup of the unborn child does not have the final say on the destiny of the child. There are countless examples of individuals with SCD who remind us that apart from the sickling gene, they possess other genes that endear them to society. Humans are known or remain unknown by their contribution to society, and not their genetic makeup. Prenatal genetic diagnosis followed by selective abortion is the equivalent of applying capital punishment to an unborn child for a “crime” the parents committed. We consider this morally and ethically unjustified.
